# Inhibitor 1 of Protein Phosphatase 1 Regulates Ca^2+^/Calmodulin-Dependent Protein Kinase II to Alleviate Oxidative Stress in Hypoxia-Reoxygenation Injury of Cardiomyocytes

**DOI:** 10.1155/2019/2193019

**Published:** 2019-12-07

**Authors:** Huiqin Luo, Shu Song, Yun Chen, Mengting Xu, Linlin Sun, Guoliang Meng, Wei Zhang

**Affiliations:** ^1^Department of Pharmacology, School of Pharmacy, Nantong University, Key Laboratory of Inflammation and Molecular Drug Target of Jiangsu Province, Nantong, Jiangsu, China; ^2^School of Medicine, Nantong University, Nantong, Jiangsu, China

## Abstract

Ca^2+^/calmodulin-dependent protein kinase II (CaMKII), regulated by inhibitor 1 of protein phosphatase 1 (I1PP1), is vital for maintaining cardiovascular homeostasis. However, the role and mechanism of I1PP1 against hypoxia-reoxygenation (H/R) injury in cardiomyocytes remain a question. In our study, after I1PP1 overexpression by adenovirus infection in the neonatal cardiomyocytes followed by hypoxia for 4 h and reoxygenation for 12 h, the CaMKII*δ* alternative splicing subtype, ATP content, and lactate dehydrogenase (LDH) release were determined. CaMKII activity was evaluated by phosphoprotein phosphorylation at Thr17 (p-PLB Thr17), CaMKII phosphorylation (p-CaMKII), and CaMKII oxidation (ox-CaMKII). Reactive oxygen species (ROS), mitochondrial membrane potential, dynamin-related protein 1 (DRP1), and optic atrophy 1 (OPA1) expressions were assessed. Our study verified that I1PP1 overexpression attenuated the CaMKII*δ* alternative splicing disorder; suppressed PLB phosphorylation at Thr17, p-CaMKII, and ox-CaMKII; decreased cell LDH release; increased ATP content; attenuated ROS production; increased mitochondrial membrane potential; and decreased DRP1 expression but increased OPA1 expression in the cardiomyocytes after H/R. Contrarily, CaMKII*δ* alternative splicing disorder, LDH release, ATP reduction, and ROS accumulation were aggravated after H/R injury with the I1PP1 knockdown. Collectively, I1PP1 overexpression corrected disorders of CaMKII*δ* alternative splicing, inhibited CaMKII phosphorylation, repressed CaMKII oxidation, suppressed ROS production, and attenuated cardiomyocyte H/R injury.

## 1. Introduction

Myocardial ischemia-reperfusion injury (MIRI) is a phenomenon wherein the myocardial function is not improved but aggravated immediately after blood perfusion is restored in the ischemic myocardium [1–4]. MIRI is often accompanied by cardiac and vascular adverse events such as arrhythmia, enlarged infarct size, persistent ventricular systolic dysfunction, or even no reflow, which seriously impair the prognosis of myocardial ischemia [5–7]. MIRI is a complex pathophysiological process involving multiple factors, such as oxygen-free radicals, calcium overload, inflammation, apoptosis, and endothelial cell homeostasis imbalance [8–10], in which excess of oxygen-free radicals is the critical factor for reperfusion injury [11]. Moreover, MIRI is a common cause of early cardiac dysfunction after cardiac surgery, which is a difficult problem to limit the treatment and prognosis of ischemic heart disease [12].

Calcium/calmodulin-dependent protein kinase II (CaMKII) is one serine-threonine protein kinase with multifunctions, which is abundant in the myocardium and other excitable tissues [13, 14]. Four isoforms of CaMKII*δ* (*α*, *β*, *γ*, and *δ*) have been found as of now. CaMKII*δ* is the most abundant subtype in the myocardium [15]. In the presence of alternative splicing, CaMKII*δ* is capable of producing three splicing variants of *δ*A, *δ*B, and *δ*C with the action of splicing factors [8–10]. Three different subtypes of CaMKII*δ* play diverse roles in the cardiovascular system. CaMKII*δ*A was critical for cardiac systolic and diastolic function [16]. CaMKII*δ*B regulated myocardial hypertrophy and progenitor cell survival in the myocardium [17]. CaMKII*δ*C was closely related to cardiomyocyte apoptosis during myocardial remodeling [18].

Besides Ca^2+^/calmodulin, CaMKII could also be activated by phosphorylation and oxidation [19–21]. Sustained excessive CaMKII activation has adverse effects on a variety of heart diseases [22, 23]. Some studies have found that CaMKII activity bloomed at the beginning of MIRI, which promoted calcium extravasation of the sarcoplasmic reticulum and aggravated myocardial dysfunction and injury [24, 25]. Alleviation of CaMKII activity is beneficial to attenuate the degree of MIRI, reduce the excessive production of reactive oxygen species (ROS) [26], and inhibit apoptosis and necrosis, which is advantageous for the recovery of the myocardial function [27]. In addition, CaMKII is also one of the key mediators for myocardial necroptosis [28]. Therefore, CaMKII is commonly regarded as the core signal in cardiomyocytes [14]. Although there were several common CaMKII inhibitors such as KN93 or AIP, the specificity and potency of these compounds limit their application due to some other effects unrelated to CaMKII inhibition [29]. Moreover, as an inhibitor of CaMKII, KN93 competitively inhibits the binding of calmodulin to kinase, but not the autonomic activity of CaMKII [29]. Altogether, CaMKII activity regulation might be a potential method to alleviate MIRI.

Protein phosphatase 1 (PP1) is regulated by intracellular calcium fluctuations in the heart to change the phosphorylation level of multiple proteins [30–33]. Studies have shown that PP1 is upregulated in the heart of patients suffering from cardiac hypertrophy, cardiac dysfunction, and heart failure [34–36]. Lack of PP1 protected against arrhythmias and myocardial hypertrophy [37]. Moreover, PP1 promoted CaMKII*δ* splicing. When PP1 increased, the ratio of CaMKII*δ* alternative splicing products could be imbalanced, resulting in an enhancement of CaMKII*δ*C variants but a reduction of CaMKII*δ*B and CaMKII*δ*A variants [33]. Therefore, the pharmacological targeting of PP1 activity is believed to be useful in therapy and interventions for a variety of cardiomyopathies such as MIRI [38–41]. As far as we know, inhibitor 1 of protein phosphatase 1 (I1PP1) is an endogenous inhibitor to decrease PP1 activity or to inhibit PP1 expression [42]. I1PP1 upregulation accelerated Ca^2+^ cycling, improved cardiac function, and alleviated cell injury [43, 44].

Therefore, hypoxia-reoxygenation (H/R) of cardiomyocytes in vitro was used to verify the effect of I1PP1 overexpression on CaMKII activity and alternative splicing. The detailed mechanism of I1PP1 overexpression against H/R injury was also explored. It is beneficial to provide a new strategy for MIRI.

## 2. Methods and Materials

### 2.1. Cell Culture and Hypoxia-Reoxygenation Injury Model

Neonatal cardiomyocytes were extracted from Sprague-Dawley (SD) rats aging 1-3 days by trypsin (Beyotime, Shanghai, China) digestion. After culturing for 2-3 days, the cells were incubated with the DMEM medium (Gibco, Carlsbad, CA) containing 1 g/L glucose and 0.5% FBS (Gibco, Carlsbad, CA) and cultured in the incubator with 94% N_2_, 1% O_2_, and 5% CO_2_ to induce hypoxia. Four hours later, the medium was changed into 5.5 g/L glucose and 10% FBS and incubated in an incubator with 5% CO_2_ and normal air. After another 12 h for reoxygenation, the cardiomyocytes were collected for further experiments. CaMKII inhibitor KN93 (1 *μ*M, MedChemExpress, Monmouth Junction, NJ) was preincubated for 1 h before hypoxia and reoxygenation.

The present experiment strictly complied with guidelines for the Care and Use of Laboratory Animals from the Institute for Laboratory Animal Research, National Research Council, Washington, D.C., National Academy Press, 2011, and any updates. The detailed protocols were also approved by a specific committee in Nantong University (NTU-20161210).

### 2.2. I1PP1 Adenovirus Infection

Rat full length of I1PP1 (Gene ID, 58977, 1 × 10^11^ PFU/mL) or vector (1 × 10^11^ PFU/mL) recombinant adenovirus solution (Hanbio Biotechnology Co., Ltd., Shanghai, China) with MOI value of 100 was infected into the cardiomyocytes. After 4 h, the adenovirus solution were washed out and DMEM medium with 10% FBS was replaced. Vector (1 × 10^11^ PFU/mL) recombinant adenovirus was applied as a negative control. Then, the cells were subjected to immunofluorescent staining, western blot, or hypoxia-reoxygenation injury.

### 2.3. RNA Interference

Three antisense oligodeoxynucleotides against rat I1PP1 mRNA (#1, 5′-AGACAATGGTTGAACATCA-3′; #2, 5′-GCAGAATCCAAACCCAAGA-3′; #3, 5′-TCAGCGTCAAGGCCAGATA′-3′) and nonspecific control siRNA (NC siRNA) were commercially obtained (RiboBio, Guangzhou, China).

After serum deprivation for 24 h, the above siRNA was transfected into the cardiomyocytes with Lipofectamine 2000 (Invitrogen, Carlsbad, CA) and subjected to hypoxia-reoxygenation injury.

### 2.4. Lactate Dehydrogenase (LDH) Measurement

The level of LDH in the medium was detected by a commercial LDH-Cytotoxic Assay Kit (Beyotime, Shanghai, China). The cardiomyocytes were cultured in a 24-well plate with a density about 80%. After I1PP1 recombinant adenovirus infection followed by hypoxia for 4 h and reoxygenation for 12 h, the supernatant of 120 *μ*L was taken into a 96-well plate after centrifugation, and 60 *μ*L of the test solution was mixed into each well. Then, the plate was placed without light for 30 min at 25°C. LDH release into the medium was calculated according to the absorbance at 490 nm, which was normalized by the value in the control group.

### 2.5. ATP Measurement

The content of ATP in the cardiomyocytes was detected by a commercial ATP Assay Kit (Beyotime, Shanghai, China). After treatment and equilibration, 100 *μ*L of a CellTiter-Lumi™ luminescence assay reagent was completely mixed with the medium. Then, the luminescence intensity was obtained with a spectrophotometer (BioTek Instruments, Inc., USA). The relative ATP level was calculated and normalized by the value in the control group.

### 2.6. ROS Measurement

After incubation with dihydroethidium (DHE, 2 *μ*M, Beyotime, Shanghai, China) at 37°C for 30 min, the superoxide production, considered as the DHE fluorescence intensity, in the cardiomyocytes was assessed with a laser confocal microscope (Leica, Wetzlar, Germany) and quantified using ImageJ software.

After incubation with MitoTracker Green (100 nM, Beyotime, Shanghai, China) and MitoSOX (5 *μ*M, YEASEN, Shanghai, China) at 37°C for 20 min, mitochondrial ROS, considered as the MitoSOX fluorescence intensity, in the cardiomyocytes was assessed with a laser confocal microscope at 490/516 nm wavelengths for MitoTracker Green and 510/580 nm wavelengths for MitoSOX, respectively, which was quantified using ImageJ software.

### 2.7. Mitochondrial Membrane Potential (*Δψ*m) Detection

After incubation with JC-1 staining solution (Beyotime, Shanghai, China) for 20 min, *Δψ*m, considered as the fluorescence intensity, in the cardiomyocytes was assessed with a laser confocal microscope at 495/519 nm wavelengths for the J-monomer and 550/570 nm wavelengths for the J-aggregates, which was quantified using ImageJ software.

### 2.8. Immunofluorescent Staining

After incubation with primary anti-optic atrophy 1 (OPA1, 1 : 50) or anti-dynamin-related protein 1 (DRP1, 1 : 50) antibodies for 12 h at 4°C, the cardiomyocytes were incubated with IgG conjugated with Cy3 or Alexa Fluor 488 (1 : 500; Beyotime, Shanghai, China) for 2 h. The protein expression which is considered as the fluorescence intensity was assessed.

### 2.9. Real-Time PCR

After extraction of total RNA with TRIzol from the cardiomyocytes, 1 *μ*L RNA sample, 2 *μ*L 5x reaction buffer, and 7 *μ*L DEPC water were mixed in the RNase-free tube. The procedure for reverse transcription was set up: incubation at 37°C for 15 minutes and 85°C for 5 s, and preservation at 4°C. Reversed cDNA was added into the SYBR Green qPCR mix for amplification (ABI, Carlsbad, CA, USA). The primer sequences (Table 1**)** of CaMKII*δ* and housekeeping mRNA were synthesized by Sangon Biotech Co., Ltd. (Shanghai, China). Quantitative real-time PCR analyses were performed three times for each group of DNA. The relative mRNA level was calculated by the comparative delta-delta cycle threshold (CT) method.

### 2.10. Western Blot

The proteins of about 50 *μ*g were separated by sodium dodecyl sulfate-polyacrylamide gel electrophoresis (SDS-PAGE) and transferred to a polyvinylidene fluoride (PVDF) membrane. Next, the membrane was blocked by 5% milk without fat for 2 h followed by incubation with primary antibodies (Table 2) [45–47] at 4°C overnight. After washing, the membrane was incubated by horseradish peroxidase- (HRP-) conjugated IgG (Beyotime, Shanghai, China) for another 2 h. Enhanced chemiluminescence (ECL, Thermo Fisher Scientific Inc., Rockford, IL, USA) was dropped on the membrane to visualize the protein bands.

### 2.11. Statistical Analysis

The data were expressed as mean ± Standard Error of the Mean (SEM), which were analyzed by one-way ANOVA followed by Bonferroni post hoc test. *P* values lower than 0.05 were regarded as a significant difference.

## 3. Results

### 3.1. CaMKII*δ* Variants Disordered in Cardiomyocytes during Hypoxia-Reoxygenation Injury

**Figure 1:**
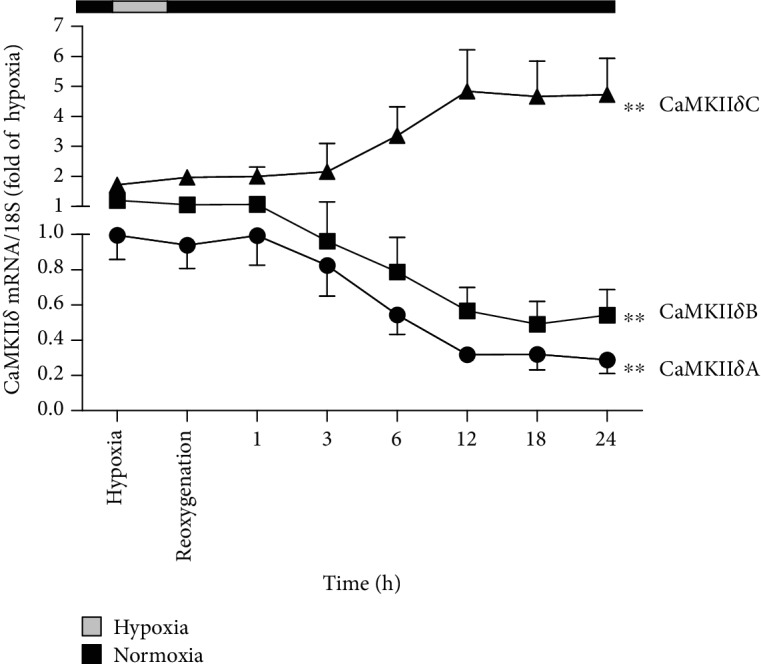
CaMKII*δ* variants disordered in cardiomyocytes during H/R injury. After culture in 94% N_2_, 1% O_2_, and 5% CO_2_ for 4 h, the cardiomyocytes were changed into 95% air and 5% CO_2_. The mRNA levels of CaMKII*δ*A, CaMKII*δ*B, and CaMKII*δ*C of the cardiomyocytes at the start of the hypoxia and different times after reoxygenation were detected by quantitative real-time PCR. 18S was serviced as a housekeeping mRNA. Plots represent the mean ± SEM; *n* = 6. Statistical significance: ^∗∗^*P* < 0.01 compared with “the start of the hypoxia.”

Because antibodies for CaMKII*δ* variants were unavailable, CaMKII*δ*A, CaMKII*δ*B, and CaMKII*δ*C expressions were measured by quantitative real-time PCR at the start of hypoxia and different times after reoxygenation. There was no significant change on CaMKII*δ* variants after hypoxia for 4 h. Both CaMKII*δ*A and CaMKII*δ*B expressions were reduced, while the CaMKII*δ*C expression increased after reoxygenation for 6 h, which was most obvious at 12 h. The data suggested that cardiomyocyte H/R injury induced a significant disorder on CaMKII*δ* variants (Figure 1).

### 3.2. I1PP1 Overexpression Reduced LDH Release but Increased ATP Level in Cardiomyocytes after H/R Injury

**Figure 2:**
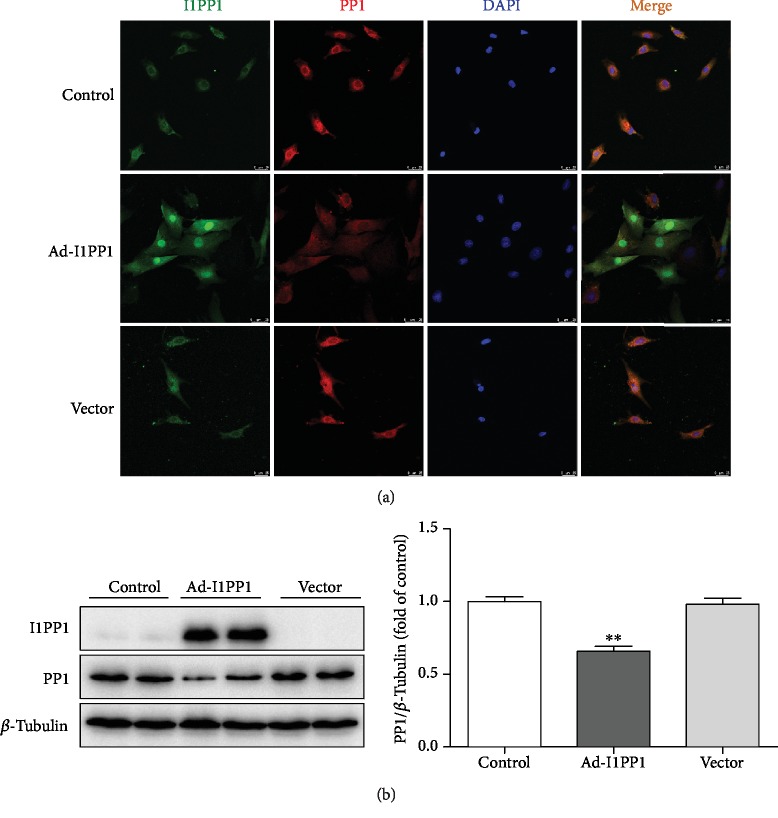
Recombinant adenovirus infection increased I1PP1 but decreased PP1 expression in cardiomyocytes. (a) After the recombinant adenovirus solution carrying the I1PP1 gene or vector was infected into the cardiomyocytes, I1PP1 and PP1 were immunofluorescence stained using Alexa Fluor 488- (green) or Cy3- (red) conjugated IgG. The nuclei were stained using DAPI (blue). Bar = 100 *μ*m. (b) Expression of the I1PP1 and PP1 protein was quantified in the cardiomyocytes by western blot. *β*-Tubulin was used as a loading control. Plots represent the mean ± SEM; *n* = 6. Statistical significance: ^∗∗^*P* < 0.01 compared with the control.

However, whether the correction of the CaMKII*δ* variant disorder was beneficial to attenuate H/R injury remains unknown. Next, the recombinant adenovirus technology was applied to induce I1PP1 overexpression in our study. PP1 antibody against PP1*α* was applied for detection of PP1 family catalytic subunits. We found that I1PP1 expression increased while PP1 expression decreased after recombinant adenovirus infection (Figure 2).

**Figure 3:**
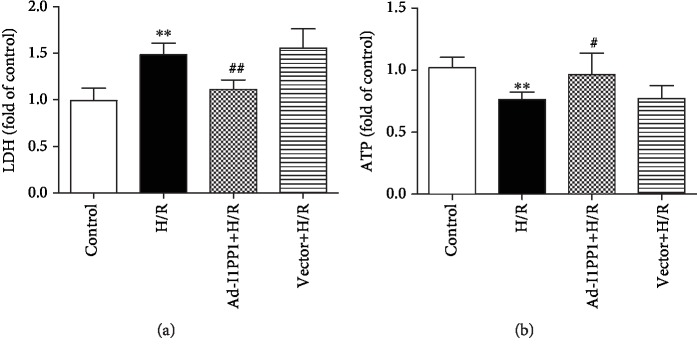
I1PP1 overexpression reduced the LDH release but increased the ATP level in cardiomyocytes after H/R injury. After infection of I1PP1 recombinant adenovirus, the cardiomyocytes were subjected to hypoxia for 4 h followed by 12 h reoxygenation. (a) LDH release in the culture medium was measured. (b) ATP level of the cardiomyocytes was measured. Plots represent the mean ± SEM; *n* = 6. Statistical significance: ^∗∗^*P* < 0.01 compared with the control; ^#^*P* < 0.05 and ^##^*P* < 0.01 compared with H/R.

After infection, hypoxia-reoxygenation was performed in the cardiomyocytes. There was more LDH in the medium after H/R, suggesting that H/R induced more serious injury. Moreover, I1PP1 overexpression in the cardiomyocytes significantly reduced LDH release (Figure 3(a)).

Dysfunction of energy metabolism is an important mechanism of myocardial ischemia-reperfusion injury [48]. ATP production was also detected to evaluate H/R injury in the cardiomyocytes. The data showed that ATP production was blocked after H/R in the cardiomyocytes, which was restored by I1PP1 overexpression (Figure 3(b)).

### 3.3. I1PP1 Overexpression Regulated CaMKII Activity and CaMKII*δ* Alternative Splicing in Cardiomyocytes after H/R Injury

**Figure 4:**
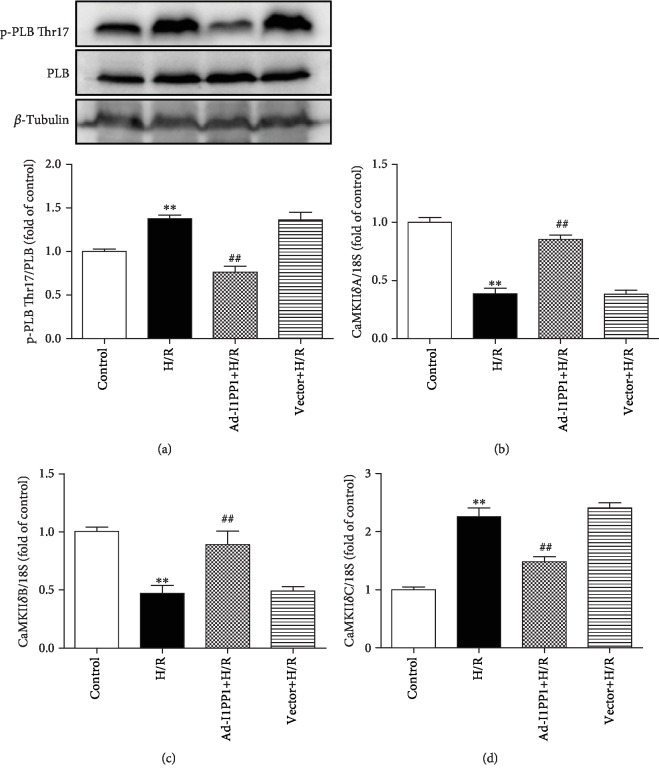
I1PP1 overexpression regulated CaMKII activity and CaMKII*δ* alternative splicing in cardiomyocytes after H/R injury. After infection of the I1PP1 recombinant adenovirus, the cardiomyocytes were subjected to hypoxia for 4 h followed by 12 h reoxygenation. (a) Expression of p-PLB Thr17 and PLB in the cardiomyocytes was quantified by western blot. *β*-Tubulin was used as a loading control. (b–d) The mRNA levels of CaMKII*δ*A, CaMKII*δ*B, and CaMKII*δ*C in the cardiomyocytes were detected by quantitative real-time PCR. 18S was serviced as a housekeeping mRNA. Plots represent the mean ± SEM; *n* = 6. Statistical significance: ^∗∗^*P* < 0.01 compared with the control; ^##^*P* < 0.01 compared with H/R.

Previous researches verified that PLB phosphorylation at Thr17 was a robust marker for CaMKII activity, which was commonly used for CaMKII activity assessment [33]. Our present study confirmed that I1PP1 overexpression significantly inhibited PLB phosphorylation at Thr17 after H/R, suggesting that CaMKII activity was weakened due to a high level of I1PP1 (Figure 4(a)). It was also noted that CaMKII*δ*A and CaMKII*δ*B splicing variants significantly decreased, while CaMKII*δ*C significantly increased after H/R. Moreover, I1PP1 overexpression increased CaMKII*δ*A and CaMKII*δ*B but decreased CaMKII*δ*C expression (Figures 4(b)–4(d)). All these data suggested that both CaMKII activity and CaMKII*δ* alternative splicing disorder were corrected by I1PP1 overexpression after H/R.

### 3.4. I1PP1 Overexpression Inhibited ROS but Elevated Mitochondrial Membrane Potential in Cardiomyocytes after H/R Injury

**Figure 5:**
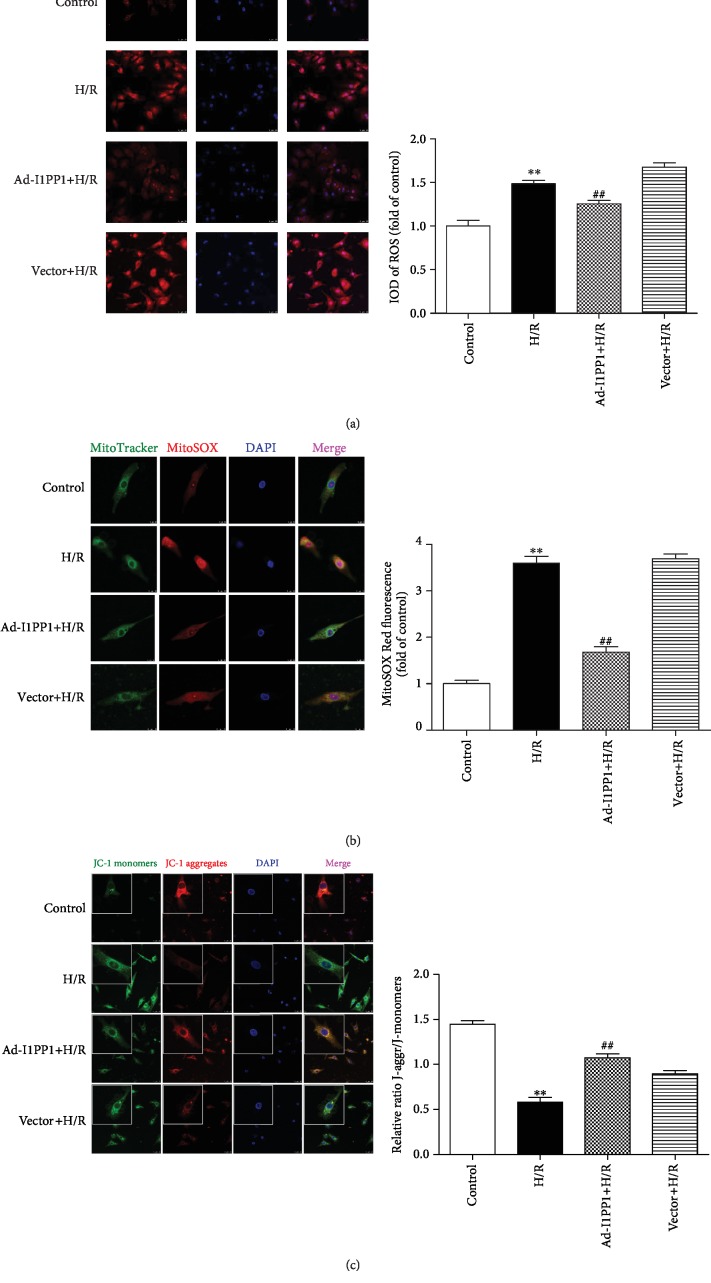
I1PP1 overexpression inhibited ROS but elevated mitochondrial membrane potential in cardiomyocytes after H/R injury. After infection of the I1PP1 recombinant adenovirus, the cardiomyocytes were subjected to hypoxia for 4 h followed by 12 h reoxygenation. (a) Superoxide production in the cardiomyocytes was detected using a fluorescence microscope with DHE fluorescent probe and quantified using ImageJ analysis software. Bar = 25 *μ*m. (b) Mitochondrial ROS was measured using MitoSOX. Mitochondrial localization of MitoSOX signal was confirmed by colocalization with MitoTracker Green and quantified using ImageJ analysis software. Bar = 10 *μ*m. (c) Mitochondrial membrane potential (*Δψ*m) was measured by JC-1 staining and quantified using ImageJ analysis software. Bar = 25 *μ*m. Plots represent the mean ± SEM; *n* = 6. Statistical significance: ^∗∗^*P* < 0.01 compared with the control; ^##^*P* < 0.01 compared with H/R.

Excessive ROS production was considered as one critical mechanism for myocardial I/R injury [49, 50]. Therefore, ROS production in the global cell and the mitochondria was detected with DHE and MitoSOX staining, respectively. The results indicated that there was stronger fluorescence intensity of DHE and MitoSOX after H/R, which was significantly attenuated by I1PP1 overexpression (Figures 5(a) and 5(b)).

Studies have shown that decrease of mitochondrial membrane potential (*Δψ*m) might lead to ROS accumulation and cell damage. Our study found that the green fluorescence intensity of JC-1 monomers, suggesting the impaired mitochondrial membrane potential, increased after H/R. Meanwhile, red fluorescence intensity of JC-1 aggregates, suggesting the normal membrane potential, decreased. Moreover, I1PP1 overexpression inhibited green but enhanced red fluorescence intensity. The data demonstrated that I1PP1 overexpression in the cardiomyocytes elevated mitochondrial membrane potential after H/R (Figure 5(c)).

### 3.5. I1PP1 Overexpression Alleviated CaMKII Oxidation and Phosphorylation in Cardiomyocytes after H/R Injury

**Figure 6:**
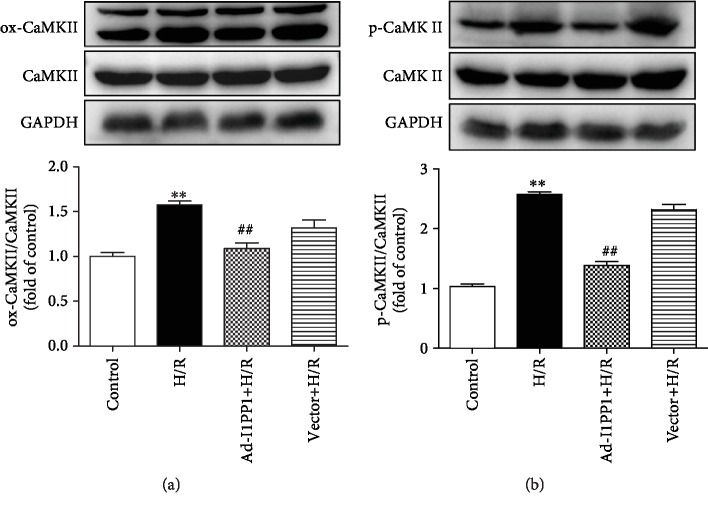
I1PP1 overexpression alleviated CaMKII oxidation and phosphorylation in cardiomyocytes after H/R injury. After infection of the I1PP1 recombinant adenovirus, the cardiomyocytes were subjected to hypoxia for 4 h followed by 12 h reoxygenation. (a) Expressions of CaMKII oxidation (ox-CaMKII) and total CaMKII in cardiomyocytes after H/R injury were quantified by western blot. GAPDH was used as a loading control. (b) Expression of CaMKII phosphorylation (p-CaMKII) and total CaMKII in cardiomyocytes after H/R injury was quantified by western blot. GAPDH was used as a loading control. Plots represent the mean ± SEM; *n* = 6. Statistical significance: ^∗∗^*P* < 0.01 compared with the control; ^##^*P* < 0.01 compared with H/R.

CaMKII oxidation and phosphorylation, as two main activation forms of CaMKII, are favorable to accelerate the deterioration of cardiac function after MIRI [28]. The study verified that both oxidation and phosphorylation of CaMKII were heightened in cardiomyocytes after H/R injury, while I1PP1 overexpression in the cardiomyocytes diminished CaMKII oxidation and phosphorylation after H/R injury (Figures 6(a) and 6(b)).

### 3.6. I1PP1 Overexpression Regulated Mitochondrial OPA1 and DRP1 Expression in Cardiomyocytes after H/R Injury

**Figure 7:**
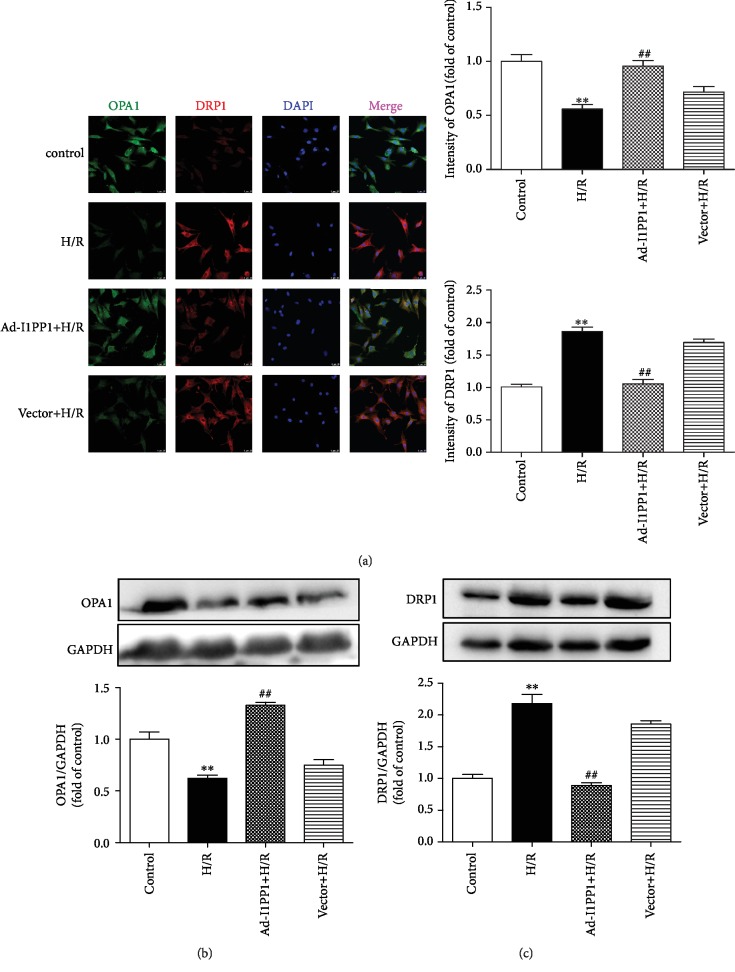
I1PP1 overexpression regulated mitochondrial OPA1 and DRP1 expression in cardiomyocytes after H/R injury. After infection of the I1PP1 recombinant adenovirus, the cardiomyocytes were subjected to hypoxia for 4 h followed by 12 h reoxygenation. (a) OPA1 and DRP1 were immunofluorescence stained using Cy3- (red) or Alexa Fluor 488- (green) conjugated IgG and quantified using ImageJ analysis software. The nuclei were stained using DAPI (blue). Bar = 10 *μ*m. (b, c) Expressions of OPA1 and DRP1 in the cardiomyocytes were quantified by western blot. GAPDH was used as a loading control. Plots represent the mean ± SEM; *n* = 6. Statistical significance: ^∗∗^*P* < 0.01 compared with the control; ^##^*P* < 0.01 compared with H/R.

OPA1 and DRP1 are mitochondrial fusion- and fission-associated proteins, respectively [51, 52]. The dynamic balance of OPA1 and DRP1 maintains mitochondrial structures and functions [53, 54]. We found that H/R injury significantly inhibited OPA1 but elevated DRP1 expression. I1PP1 overexpression in the cardiomyocytes increased OPA1 but decreased DRP1 after H/R injury, which was beneficial to maintaining the balance of OPA1 and DRP1 (Figures 7(a)–7(c)).

### 3.7. I1PP1 Overexpression Combining with CaMKII Inhibitor KN93 Attenuated Oxidative Stress in Cardiomyocytes after H/R Injury

**Figure 8:**
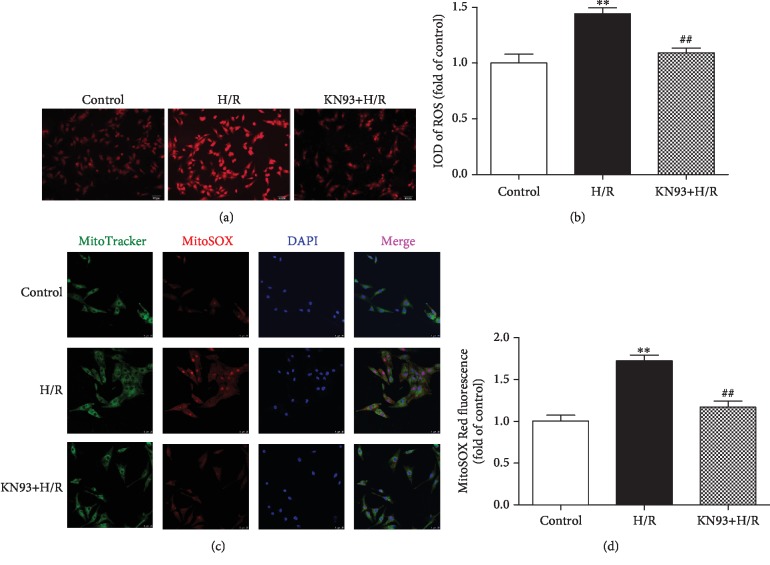
KN93 attenuated oxidative stress in cardiomyocytes after H/R injury. After CaMKII inhibitor KN93 (1 *μ*M) preadministration for 1 h, the cardiomyocytes were subjected to hypoxia for 4 h followed by 12 h reoxygenation. (a, b) Superoxide production in the cardiomyocytes was detected using a fluorescence microscope with a DHE fluorescent probe and quantified using ImageJ analysis software. Bar = 100 *μ*m. (c, d) Mitochondrial ROS was measured using MitoSOX. Mitochondrial localization of the MitoSOX signal was confirmed by colocalization with MitoTracker Green and quantified using ImageJ analysis software. Bar = 10 *μ*m. Plots represent the mean ± SEM; *n* = 6. Statistical significance: ^∗∗^*P* < 0.01 compared with the control; ^##^*P* < 0.01 compared with H/R.

In order to confirm the above protective effect of I1PP1 overexpression against oxidative stress during H/R injury was ascribed to CaMKII inhibition, the global cellular ROS and mitochondrial ROS were further measured. The study found that CaMKII inhibitor KN93 preadministration significantly inhibited ROS accumulation after H/R injury (Figures 8(a)–8(d)).

**Figure 9:**
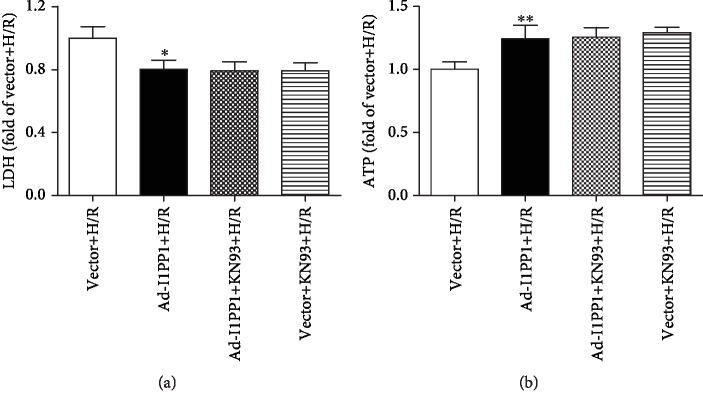
Effects of I1PP1 overexpression and KN93 on LDH release and ATP level in cardiomyocytes after H/R injury. After infection of the I1PP1 recombinant adenovirus, the cardiomyocytes with or without CaMKII inhibitor KN93 incubation for 1 h were subjected to hypoxia for 4 h followed by 12 h reoxygenation. (a) LDH release in the culture medium was measured. (b) ATP level of the cardiomyocytes was measured. Plots represent the mean ± SEM; *n* = 6. Statistical significance: ^∗^*P* < 0.05 and ^∗∗^*P* < 0.01 compared with vector+H/R.

**Figure 10:**
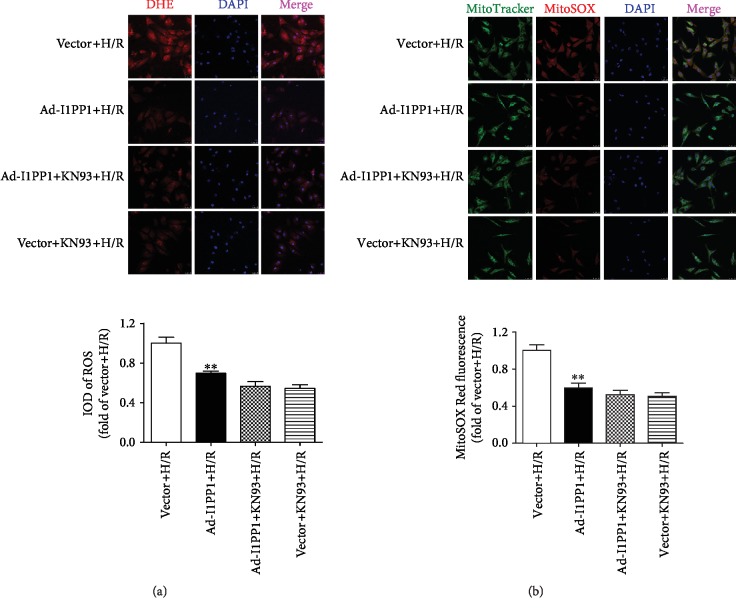
Effects of I1PP1 overexpression and KN93 on oxidative stress in cardiomyocytes after H/R injury. After infection of the I1PP1 recombinant adenovirus, the cardiomyocytes with or without CaMKII inhibitor KN93 incubation for 1 h were subjected to hypoxia for 4 h followed by 12 h reoxygenation. (a) Superoxide production in the cardiomyocytes was detected using a fluorescence microscope with DHE fluorescent probe and quantified using ImageJ analysis software. Bar = 25 *μ*m. (b) Mitochondrial ROS was measured using MitoSOX. Mitochondrial localization of the MitoSOX signal was confirmed by colocalization with MitoTracker Green and quantified using ImageJ analysis software. Bar = 25 *μ*m. Plots represent the mean ± SEM; *n* = 6. Statistical significance: ^∗∗^*P* < 0.01 compared with vector+H/R.

**Figure 11:**
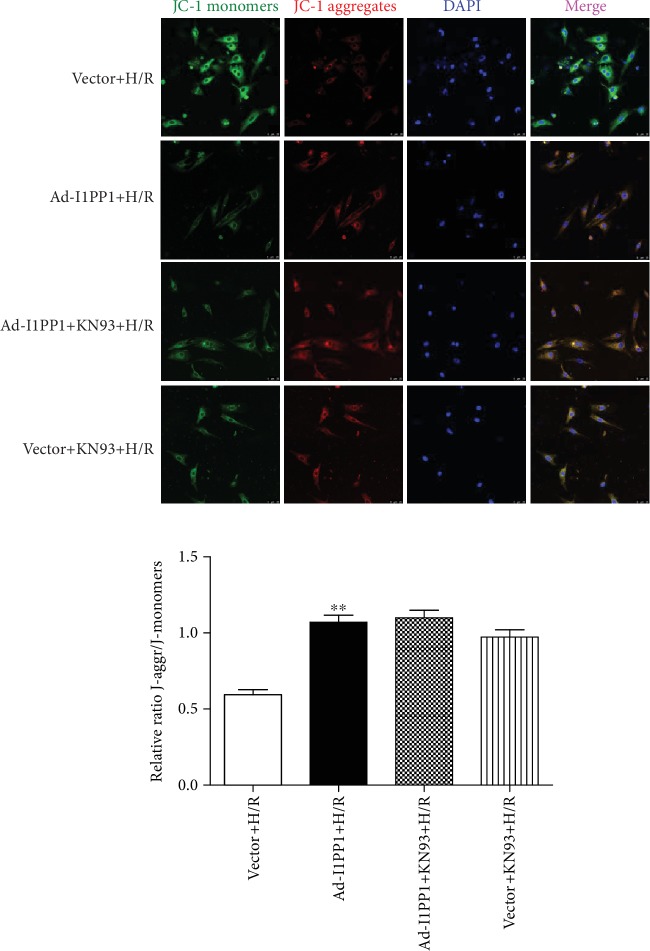
Effects of I1PP1 overexpression and KN93 on mitochondrial membrane potential in cardiomyocytes after H/R injury. After infection of the I1PP1 recombinant adenovirus, the cardiomyocytes with or without CaMKII inhibitor KN93 incubation for 1 h were subjected to hypoxia for 4 h followed by 12 h reoxygenation. Mitochondrial membrane potential (*Δψ*m) was measured by JC-1 staining and quantified using ImageJ analysis software. Bar = 25 *μ*m. Plots represent the mean ± SEM; *n* = 6. Statistical significance: ^∗∗^*P* < 0.01 compared with vector+H/R.

Moreover, both I1PP1 overexpression and I1PP1 overexpression combining with KN93 similarly decreased LDH release (Figure 9(a)) and enhanced ATP production (Figure 9(b)), weakened ROS production (Figure 10), and increased mitochondrial membrane potential (Figure 11) in cardiomyocytes after H/R. Furthermore, no significant difference appeared between the above two groups. These data indicated that I1PP1 overexpression alleviated cell damage, inhibited ROS production, and elevated mitochondrial membrane potential in cardiomyocytes after H/R injury via inhibition of CaMKII activation.

### 3.8. I1PP1 Knockdown Exacerbated CaMKII*δ* Alternative Splicing Disorder, Cell Injury, and Oxidative Stress in Cardiomyocytes after H/R Injury

**Figure 12:**
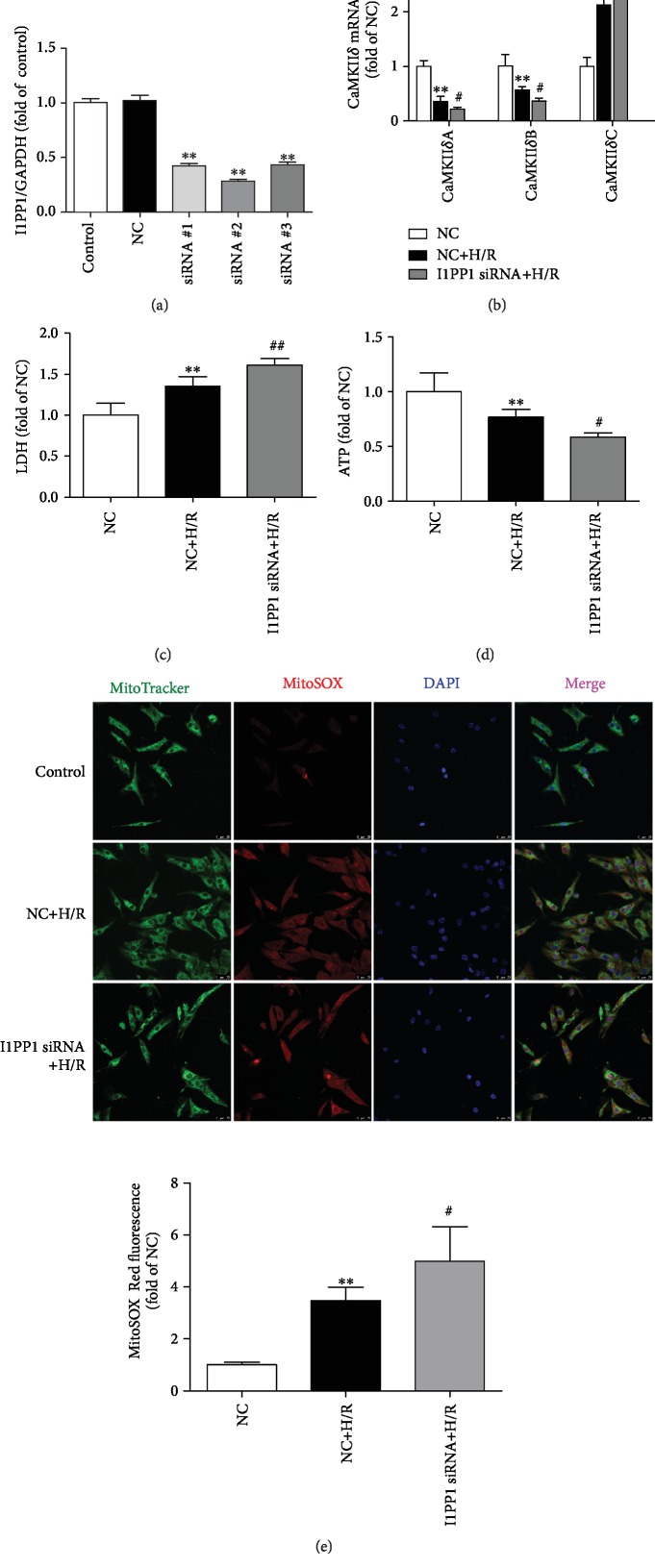
Effects of I1PP1 knockdown on CaMKII*δ* alternative splicing disorder, cell injury, and oxidative stress in cardiomyocytes after H/R injury. (a) After I1PP1 siRNA or NC siRNA was transfected into neonatal rat cardiomyocytes, expressions of I1PP1 in the cardiomyocytes were quantified by western blot. GAPDH was used as a loading control. (b) After I1PP1 siRNA or NC siRNA was transfected into neonatal rat cardiomyocytes, cells were subjected to hypoxia for 4 h followed by 12 h reoxygenation. The mRNA levels of CaMKII*δ*A, CaMKII*δ*B, and CaMKII*δ*C of the cardiomyocytes after reoxygenation were detected by quantitative real-time PCR. 18S was serviced as a housekeeping mRNA. (c) LDH release in the culture medium was measured. (d) ATP level of the cardiomyocytes was measured. (e) Mitochondrial ROS was measured using MitoSOX. Mitochondrial localization of the MitoSOX signal was confirmed by colocalization with MitoTracker Green and quantified using ImageJ analysis software. Bar = 25 *μ*m. Plots represent the mean ± SEM; *n* = 6. Statistical significance: ^∗∗^*P* < 0.01 compared with NC; ^#^*P* < 0.05 and ^##^*P* < 0.01 compared with NC+H/R.

Contrarily, we also assessed the effect of I1PP1 knockdown with siRNA technology in cardiomyocytes under H/R injury. We found that all three siRNA significantly reduced I1PP1 expression, and there was the least expression of I1PP1 after siRNA#2 transfection. Therefore, siRNA#2 was applied in a further study (Figure 12(a)). Next, we found that the CaMKII*δ* alternative splicing disorder (Figure 12(b)), LDH release (Figure 12(c)), ATP reduction (Figure 12(d)), and ROS accumulation (Figure 12(e)) in cardiomyocytes were aggravated after H/R in the cardiomyocytes with I1PP1 knockdown. However, there was no evidence regarding CaMKII activation in neonatal cardiomyocytes after I1PP1was knocked down. Altogether, I1PP1 knockdown exacerbated the CaMKII*δ* alternative splicing disorder, cell injury, and oxidative stress in neonatal cardiomyocytes after H/R injury.

## 4. Discussion

Although reperfusion is essential for blood flow recovery in the myocardium, it might result in serious damage to the heart during myocardium ischemia and reperfusion. Thus, how to attenuate MIRI has been a very difficult problem in the clinic.

CaMKII is capable of integrating *β*-adrenergic, ROS, Gq-coupled receptors, hyperglycemia, and proapoptotic cytokine signals to induce oxidative stress in the myocardium [14]. The excessive activation of CaMKII exacerbates cardiomyocyte damage and accelerates the progression of cardiovascular disease. Previous research indicated that CaMKII was crucial in the pathogenesis of MIRI [55–59], and CaMKII*δ* was the most vital isoform in the myocardium. Our present results showed that hypoxia-reoxygenation caused a disorder of CaMKII*δ* alternative splicing in the cardiomyocytes, which was characterized by decreased expression of the *δ*A and *δ*B subtypes but increased expression of *δ*C. On the other hand, levels of ox-CaMKII and p-CaMKII, as well as expression of p-PLB Thr17 representing CaMKII activity, also increased. Previous study found that PLB phosphorylation increased in the heart of I1PP1 transgenic mice in the basal state. I1PP1 elevated PLB phosphorylation after global ischemia for 40 min followed by reperfusion, whereas there were no differences at 60 min postreperfusion [43]. I1PP1 also increased PLB phosphorylation in the heart of pigs in the basal state [60]. In the present study, we found that I1PP1 significantly inhibited PLB phosphorylation after H/R in cardiomyocytes. The divergent effects on PLB phosphorylation may be explained by different types of cells or animals used in such studies, different ischemia or reperfusion times, different times for measurement, or a combination of these factors. To our knowledge, ROS oxidizes the methionine residue site of Met281/282 to promote CaMKII oxidation. CaMKII was able to be phosphorylated by itself. However, whether restoring the above abnormalities of CaMKII is able to alleviate H/R injury is not well known.

PP1 is able to alter the splicing factor phosphorylation and regulate CaMKII alternative splicing in the heart. A previous study found that hypoxia (but without reoxygenation) decreased the activity of PP1 in the cardiomyocytes [61], in the skeletal muscle of frogs [62], in the eukaryotic cells [63], and in the hippocampus [64]. A previous study found that enhanced PP1 activity was involved in Ca^2+^ cycling, augmenting during H/R in the adult heart [43]. Moreover, previous research found that inducible overexpression of I1PP1 enhanced basal cardiac function and protected against ischemia-reperfusion injury [43], which was consistent with our results that I1PP1 overexpression attenuated cardiomyocyte H/R injury. Our research also confirmed that I1PP1 corrected the disorder of CaMKII*δ* splicing, reduced LDH release, but enhanced ATP production to alleviate cell injury. Contrarily, I1PP1 knockdown exacerbated the CaMKII*δ* alternative splicing disorder and aggravated cell injury after H/R injury. The protective effect of I1PP1 overexpression may be ascribed to the regulation of CaMKII alternative splicing and inhibition of CaMKII activity and ox-CaMKII as well as p-CaMKII. These data indicated that I1PP1 had a negative regulatory effect on H/R injury of cardiomyocytes through CaMKII regulation.

The protective mechanism of the attenuating effect on H/R damage by I1PP1 overexpression might be related to ROS inhibition. As far as we know, excessive ROS usually resulted in several types of pathological damage including myocardial hypertrophy [65, 66], liver injury or fibrosis [67, 68], pulmonary damage [69], and neuronal apoptosis [70]. The unsaturated fatty acids were vital for maintaining mitochondrial integrity and function, which is extremely susceptible to oxidative injury. The increase of oxidized unsaturated fatty acids directly impaired the function of the mitochondrial electron transport chain, thereby further promoting more ROS generation in mitochondria [71]. During H/R, ROS also induced the transformation of mitochondrial permeability by opening several small pores in the mitochondria immediately at the beginning of reperfusion [72], which resulted in mitochondrial matrix swelling and outer membrane integrity destruction. Besides, mitochondrial damage also directly blocks the production and storage of cellular ATP. Therefore, maintaining normal structure and function of mitochondria is critical for cell survive [73, 74]. Our present results showed that H/R decreased the mitochondrial membrane potential and promoted ROS accumulation. After I1PP1 successfully inhibited PP1 expression, the CaMKII pathway, cell damage, ATP production, and mitochondrial membrane potential as well as ROS accumulation were restored significantly. Moreover, CaMKII-specific inhibitor KN93 was further applied to clarify the causal relationship between negative regulation of CaMKII and the protective effect on cardiomyocytes. Our data indicated that KN93 was capable of attenuating both global intracellular and mitochondrial ROS after H/R. We also found that I1PP1 overexpression with or without CaMKII inhibition by KN93 similarly decreased LDH release, weakened ROS production, and increased mitochondrial membrane potential in cardiomyocytes after H/R. Altogether, it suggested that I1PP1 overexpression attenuated cardiomyocyte injury under stress conditions via inhibiting CaMKII activation.

Dynamic fusion and fission is beneficial to maintaining mitochondrial structure and function to appropriately respond to frequent changing environmental conditions [75]. OPA1 localizes on the mitochondrial inner membrane (IMM) and membrane gap to control IMM fusion and sputum structure [76]. OPA1 deficiency promoted mitochondrial fragmentation and abnormal sputum remodeling to suppress respiratory ability and inhibit mitochondria-dependent cell metabolism [77, 78]. Besides, OPA1 also prevented cytochrome C redistribution and release to inhibit cell injury [54]. Contrarily, DRP1 has been verified to mediate mitochondrial fission [79]. In our present study, hypoxia-reoxygenation caused the imbalance of OPA1 and DRP1 in cardiomyocytes, which would cause fatal damage to the morphology and function of mitochondria. Overexpression of I1PP1 reversed the imbalance of proportions between OPA1 and DRP1 which is normal to maintain the mitochondrial homeostasis.

However, there are several limitations in our present study. Firstly, the use of human cardiomyocytes and measurement of CaMKII levels will greatly benefit extending the significance of the study, which will directly demonstrate the real impact of I1PP1 overexpression on cardiomyocytes for humans. However, human cardiomyocytes are unavailable in the present study. Secondly, due to the experimental strategy that mainly relies on the overexpression of I1PP1, it is unclear whether endogenous I1PP1 has a significant role under H/R injury in neonatal cardiomyocytes. The knockdown approach was performed in addition to overexpression of I1PP1. However, detection of CaMKII activity and CaMKII phosphorylation as well as PP1 levels was unavailable in the present study, which will be a great benefit to validate the effect and mechanism of I1PP1 on H/R injury in cardiomyocytes.

**Figure 13:**
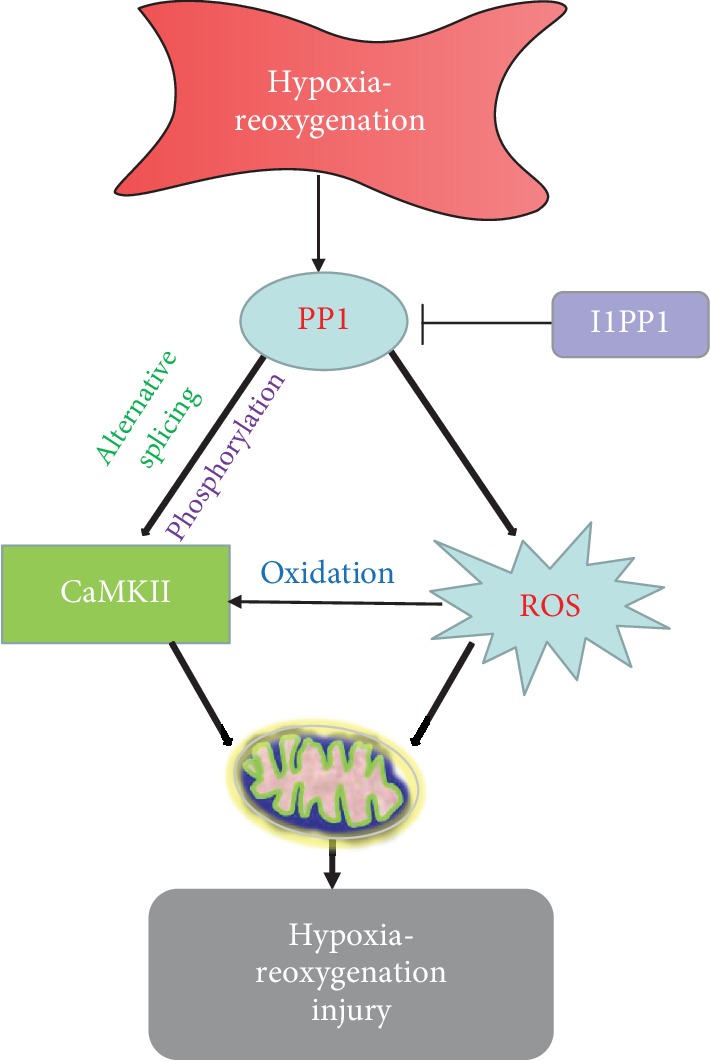
I1PP1 overexpression corrected CaMKII*δ* alternative splicing disorders, inhibited CaMKII phosphorylation, repressed CaMKII oxidation, suppressed ROS production, and attenuated hypoxia-reoxygenation injury in the cardiomyocytes.

Collectively, I1PP1 overexpression corrected disorders of CaMKII*δ* alternative splicing, inhibited CaMKII phosphorylation, repressed CaMKII oxidation, suppressed ROS production, and attenuated H/R injury in the cardiomyocytes (Figure 13). The present study is helpful to provide new biological targets for prevention and treatment of myocardial ischemia-reperfusion injury in the clinic.

## Figures and Tables

**Table 1:** The sequences of the primers for real-time PCR.

Gene	Forward primers	Reverse primers
CaMKII*δ*A	5′-CGAGAAATTTTTCAGCAGCC-3′	5′-ACAGTAGTTTGGGGCTCCAG-3′
CaMKII*δ*B	5′-CGAGAAATTTTTCAGCAGCC-3′	5′-GCTCTCAGTTGACTCCATCATC-3′
CaMKII*δ*C	5′-CGAGAAATTTTTCAGCAGCC-3′	5′-CTCAGTTGACTCCTTTACCCC-3′
18S	5′-AGTCCCTGCCCTTTGTACACA-3′	5′-CGATCCGAGGGCCTCACTA-3′

**Table 2:** The primary antibodies for western blot.

Protein	Dilution	Company
I1PP1	1 : 5000	Abcam, Cambridge, UK
OPA1	1 : 1000	Abcam, Cambridge, UK
CaMKII	1 : 1000	Abcam, Cambridge, UK
PP1	1 : 1000	Santa Cruz Biotechnology, Santa Cruz, CA, USA
PLB	1 : 1000	Santa Cruz Biotechnology, Santa Cruz, CA, USA
p-PLB Thr17	1 : 1000	Santa Cruz Biotechnology, Santa Cruz, CA, USA
DRP1	1 : 1000	Cell Signaling Technology, Danvers, MA, USA
p-CaMKII	1 : 1000	Thermo Fisher Scientific, Rockford, IL, USA
ox-CaMKII	1 : 1000	Millipore, Billerica, MA, USA
GAPDH	1 : 5000	Sigma-Aldrich, St. Louis, MO, USA
*β*-Tubulin	1 : 3000	CMCTAG, Milwaukee, WI, USA

## Data Availability

The data used to support the findings of this study are available from the corresponding authors upon request.
